# Unprecedented 2020 coral bleaching reveals unexpected taxa-specific responses in the central Red Sea

**DOI:** 10.1371/journal.pone.0331235

**Published:** 2025-09-09

**Authors:** Natalie Dunn, Fabio Marchese, Catherine S. McFadden, Laura Macrina, Marta Ezeta Watts, Megan K. B. Nolan, Francesca Giovenzana, Francesca Benzoni

**Affiliations:** 1 Marine Science Program, Biological and Environmental Science and Engineering Division (BESE), King Abdullah University of Science and Technology (KAUST), Thuwal, Kingdom of Saudi Arabia; 2 KAUST Red Sea Research Center, King Abdullah University of Science and Technology (KAUST), Thuwal, Kingdom of Saudi Arabia; 3 Department of Biology, Harvey Mudd College, Claremont, California, United States of America; 4 KAUST Ali I. Al-Naimi Petroleum Engineering Research Center, King Abdullah University of Science and Technology (KAUST), Thuwal, Kingdom of Saudi Arabia; University of the Ryukyus, JAPAN

## Abstract

Sea surface temperature of the Red Sea has increased by up to 0.45 °C per decade over the last 30 years, and coral bleaching events are becoming more frequent. A reef bleaching event was observed in October 2020, whereby some parts of the Red Sea experienced more than 12 °C-weeks. The study sites spanned nearly three degrees of latitude along the central Saudi Arabian Red Sea and were surveyed via structure-from-motion photogrammetry in October 2020 during the bleaching event and again in October 2022 to track the fate of the coral colonies. The *in situ* temperatures in 2020 ranged from 31.9 °C to 32.7 °C, and overall, 65% of the colonies exhibited some bleaching. Nearly half of the colonies exhibited partial or complete mortality in 2022, although 18% exhibited complete mortality. Approximately 27% of the colonies presented no visible change in coloration over the study period, whereas 21% presented recovery over the two years. *Porites, Montipora, Pocillopora*, and *Stylophora* were classified as winners, whereas *Acropora, Goniastrea*, Xeniidae, and *Sclerophytum* were classified as losers. At the time of this study, this research was the first to assess the longest-term changes in coral colonies following a major reef bleaching event in the central Saudi Arabian Red Sea. The results suggest that the 2020 bleaching event may be the most severe event on record for the region at the time of the study, and our data underscore the need for enhanced monitoring of corals and environmental data to better understand coral reef ecosystem resilience in a historically data scarce region.

## Introduction

Shallow-water benthic coral communities are highly dynamic systems and are in a constant cycle of disturbance and recovery [1–4]. Biological and physical processes across multiple spatial and temporal scales, such as predation, storms, disease, and sea level change, shape community dynamics on coral reefs and govern growth and mortality regimes in corals [1,5–8]. Coral mortality dynamics may include both whole-colony (complete) and partial (incomplete) mortality [9–14]. However, investigating these dynamics across large spatial and temporal scales is challenging, and few studies have examined noncatastrophic disturbance regimes to estimate background mortality rates on reefs between 5 m and 10 m deep. For example, mortality rates as part of the dynamic equilibrium in the absence of acute stressors or ephemeral events (background mortality) were estimated to be up to 29% of colonies (at least 80 cm^2^ in area) over a 3.4 year period in Heron Island [15], and 26% of colonies greater than 30 cm in diameter suffered some mortality over a span of five years in Curaçao [16]. The prevalence of partial mortality was estimated to be less than 5% for Florida reefs [17] and Australia’s Coral Sea Marine Park [18]. In the Indo-Pacific region, background (whole-colony) mortality was estimated to be less than 8% of colonies per annum [19], and rates of complete mortality of 20% and 30% were classified as moderate and high, respectively [20]. Background mortality in the Red Sea was estimated to be relatively low, whereby dead coral represented an average of 7% of the benthic cover [21,22].

While cycles of disturbances and recovery are ongoing in shallow-water corals, increasing anthropogenic influences can increase the frequency, duration, and intensity of some of these events, reducing the time and opportunity for recovery [23–28]. One such example is coral bleaching. In recent decades, climate change has led to warmer ocean temperatures [14,23,29–33]. This source of potential thermal stress together with elevated solar irradiance can lead to the breakdown of the coral-symbiont association in zooxanthellate organisms [12,34–37] and expulsion of symbiont cells, causing the coral to lose their soft tissue coloration and appear bleached [23,35,38–41]. The thermal threshold for coral bleaching varies with the intensity, duration, and recurrence of thermal stress, as well as the geographic region and taxa involved [23,38,39,42]. Nevertheless, it has been suggested that temperatures only 1–2 °C greater than the summer maxima can lead to coral bleaching [38]. A bleached coral may suffer complete or partial mortality depending on a number of factors, including colony size and species affiliation, environmental conditions, and the intensity of the bleaching event [12,38,42]. Various processes of physical and bioerosion then abrade the dead skeleton, which ultimately results in a loss in structural complexity important for reef biodiversity [43–48]. However, if environmental and biotic conditions allow, the coral may be recolonized by endosymbionts and exhibit recovery, although fitness may be reduced [13,20,42,49,50].

The differential susceptibility of corals to bleaching and bleaching associated mortality has been examined across various temporal and spatial scales. For example, long-term impacts from 2 to 14 years after a thermal stress event have been studied in the Pacific Ocean [51–53], Indian Ocean [13,54], and Arabian Gulf [55]. Within two years postbleaching, these studies showed that thin-tissued, branching and plating scleractinian colonies with relatively fast growth rates and other weedy traits, such as *Acropora*, *Pocillopora*, and *Stylophora*, declined in relative abundance, whereas thick-tissued, massive and encrusting growth forms, such as *Porites*, did not bleach or recovered from bleaching [13,51,52,55,56,57]. Corals that exhibit higher rates of mortality than background mortality have been termed losers, whereas corals that do not bleach or recover from bleaching have been termed winners [20,51,52,58]. This differential susceptibility to bleaching and mortality across taxa can reduce species richness [59–61], shift community compositions from branching to massive growth forms [51,60,62,63], favor more thermally tolerant taxa, and alter size-frequency distributions toward smaller colony dominance [64]. However, by three years postbleaching, certain branching corals, such as *Acropora* and *Montipora*, became winners as a result of rapid regrowth and/or recruitment [52] or acclimation due to shifts toward more thermally-tolerant Symbiodiniaceae communities [65,66], although species richness may remain low [55]. Alternatively, in some cases, soft corals [67–71] and corallimorpharians [72–74] have been observed to dominate communities following disturbances that specifically impact hard corals whereby their competitive advantages over hard corals, including high fecundity, multiple modes of dispersal, and ability to inhibit larval recruitment of hard corals [75–77], may enable them to establish dominance. *Sclerophytum* (and *Sinularia,* see [78] for a revision), was the most resistant soft coral to bleaching following long-term surveys in Japan, and while *Lobophytum* and *Sarcophyton* initially decreased following bleaching, both recovered to prebleaching levels of relative abundance after 12 years [52]. Some species of *Sclerophytum* have also been shown to monopolize large areas in the Great Barrier Reef, while there have been occasional monospecific stands of *Xenia*, *Lobophytum* [68], and *Clavularia* [72] and reports of soft-coral dominated communities overall in the Indo-Pacific [79]. However, compared with scleractinians, only a limited number of studies have investigated bleaching in octocorals, and observations have been largely anecdotal [51,80,81].

Previous coral bleaching studies have estimated population-level metrics to investigate the long-term impacts of bleaching on coral communities utilizing various *in situ* survey techniques, including photoquadrats, random quadrats, and line-intercept transects (LIT) [13,51,52,55]. However, fate tracking of individual colonies enables the investigation of patterns of coral bleaching, mortality, and recovery on a relatively fine scale [82–86]. While tagging and tracking individual colonies *in situ* over time can be challenging due to complex colony-scale dynamics, including partial mortality, fission, and fusion [86], structure-from-motion (SfM) photogrammetry has enabled high-resolution, large-scale benthic surveys that facilitate tracking individual colonies over time [87–91]. SfM generates a complete three-dimensional reconstruction of the reef, which is then projected as a two-dimensional orthomosaic [47,48,90]. This method is comparable to traditional *in situ* surveys [90] and holds great potential for monitoring coral colonies and reef structural complexity over time, with the advantage of precise location tracking and ability to cover large areas. However, consistent, standardized protocols in terms of imagery acquisition and reference points are needed to enable accurate reef structural comparisons across years. Nevertheless, the high-resolution orthomosaics provide a permanent photo-record of the benthic community and thus an opportunity for long-term monitoring, with data that can be verified and revisited for future analyses.

Unlike the previously discussed Pacific Ocean, Indian Ocean, and Arabian Gulf [13,51–55], assessing mortality of coral colonies following a bleaching event in the central Saudi Arabian Red Sea has been limited [92,93]. Shallow-water coral communities in the central Red Sea exhibit high levels of biodiversity and endemism and harbor some of the most thermally tolerant corals in the world [41,94–99]. However, as these corals live at their thermal maxima, the region is not immune to bleaching events [41,63,93]. During the 1997–1998 El Niño event [23,100–102], Degree Heating Weeks (DHWs), which is a measure of cumulative heat stress calculated by the number of weeks in which sea surface temperature exceeds 1 °C above the maximum monthly mean over three months [103], reached 9 °C-weeks in the central Saudi Arabian Red Sea, according to the National Oceanic and Atmospheric Administration (NOAA) Coral Reef Watch [104]. Up to 65% of corals on nearshore reefs in the central Red Sea bleached during this time [22], with an estimated 30% mortality by 1999 based on timed swim SCUBA surveys [92]. Another global bleaching event occurred in 2010 [105,106], and DHWs reached 10 °C-weeks in the central Red Sea [107]. Following this event, approximately 74% of hard corals on inshore reefs and 14% on offshore reefs in the region bleached, and the bleaching prevalence ranged from 13% to 82% across sites, as estimated by the LIT method on SCUBA [93]. After seven months, inshore reefs exhibited reduced species richness and a loss of faster-growing, branching corals, including *Acropora* and *Pocillopora* [93]. The longest global bleaching event on record to date occurred in 2015–2016 in connection with an El Niño thermal anomaly [105,106,108,109], however DHWs only reached a maximum of 1.7 °C-weeks in Thuwal, Saudi Arabia [63,110]. Similar to the 2010 reef bleaching event, shallower inshore reefs exhibited a greater incidence of bleaching than offshore reefs did in 2015, likely due to reduced circulation, a lower heat budget, and proximity to terrestrial stressors [63,93]. The incidence of bleaching ranged from 0.6% to 67% of hard corals across sites, as estimated using the LIT method on SCUBA [63]. Red Sea reefs again exhibited common patterns in 2015, such as high bleaching rates in branching corals, *Acropora* and *Pocillopora*, although postbleaching mortality was not investigated [63]. The prevalence of bleaching for both the 2010 and 2015 events across zooxanthellate reef-building corals *Acropora, Montipora*, *Echinopora, Goniastrea, Pocillopora*, and *Porites* is summarized in Table 1 [63,93], yet postbleaching mortality in the region remains largely understudied.

**Table 1:** Coral bleaching susceptibility across taxa during previous bleaching events in the central Saudi Arabian Red Sea. Data from 2015 and 2010 were obtained from Monroe et al. 2018 and Furby et al. 2013, respectively.

Taxon	% cover bleached	
	2015	2010
Acroporidae		35
*Acropora*	38	
*Montipora*	35	
Merulinidae		38
*Echinopora*	20	
*Goniastrea*	35	
Pocilloporidae (*Pocillopora*)	18	18
Poritidae (*Porites*)	35	35

More recently, many coral reef areas worldwide experienced significant thermal stress in 2020, and the Great Barrier Reef, Indonesia, the Western Pacific, and the Caribbean were identified as bleaching alert areas [111]. In addition, temperatures exceeded 12 °C-weeks in the central Red Sea between Rabigh and Yanbu, Saudi Arabia, in October 2020 [112]. While there is a paucity of data regarding coral bleaching observations in 2020, likely due to the coronavirus pandemic 2019 (COVID-19), unprecedented bleaching prevalence of approximately 45% to 80% of coral cover in the South China Sea were estimated using the LIT method [113]. Similarly, a marine heatwave was reported in the Great Barrier Reef [114], and aerial surveys estimated that between 20% and 60% of reefs [115] and approximately 50% of corals at One Tree Island [116] exhibited severe bleaching, although some areas exhibited bleaching of up to 98% of coral cover [117].

Importantly, a fourth global coral bleaching event was recognized in 2023–2024 [118–120], and as bleaching events are predicted to become both more frequent and more severe [23,28,32,121], it is imperative to better understand long-term, taxon-specific responses to bleaching, and high-resolution SfM orthomosaics can improve our ability to fate track individual colonies over a long period of time. The aims of this study are to (1) describe the thermal anomaly in the central Saudi Arabian Red Sea in October 2020 using remotely sensed sea surface temperature (SST) and DHW data and *in situ* temperature data and (2) fate track scleractinian and octocoral colonies to describe mortality and survival following bleaching across taxa via long-term monitoring using SfM-generated orthomosaics for the first time in the Red Sea.

## Methodology

### Study sites

**Fig 1:**
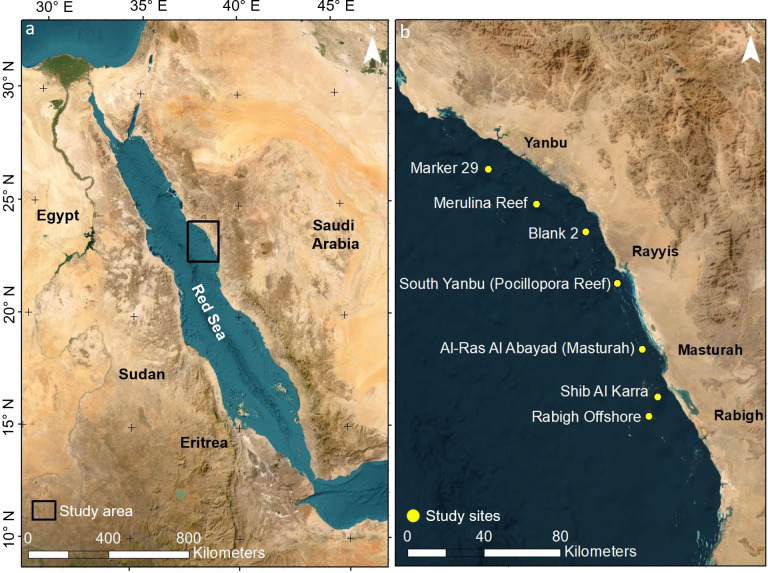
Study sites in the central Saudi Arabian Red Sea. (a) Map of the Red Sea showing the location of the study region (black polygon). (b) Study sites between Rabigh and Yanbu surveyed in 2020 and 2022. Basemap satellite images accessed from World Imagery ESRI Tile Layer. Credits: Esri, Maxar, GeoEye, Earthstar Geographics, CNES/Airbus DS, USDA, USGS, AeroGRID, IGN, and the GIS User Community.

In October 2020, during the bleaching event, seven offshore reefs spanning more than 165 km of coastline between Rabigh and Yanbu, Saudi Arabia, were opportunistically selected and surveyed during the KAUST-Yanbu 2020 Expedition onboard M/Y Dream Island (Fig 1). Sites were resurveyed two years later, in October 2022, during the KAUST-Yanbu 2022 Expedition onboard the M/Y Dream Master. One transect per site was installed on the exposed side of each reef along the depth contour, between 5 and 10 m deep, where bleaching was previously most evident in the region [63,93]. The target dimensions of each transect in the field were 30 m long by 5 m wide, following the protocol developed by Fukunaga et al. [122,123] in Hawaii. The transects were intentionally longer than those used in previous bleaching (10 m) [63,93] and benthic surveys (20 m) [124] in the central Red Sea to (1) capture the patchy nature of Red Sea coral communities [125] and (2) anticipate that the reconstructed digital area would be smaller due to high reef sinuosity and uncertainty of overlap between years.

### Image acquisition and model generation

A SCUBA diver collected a series of overlapping images for each transect by swimming slowly in a lawnmower pattern 1 m above the substrate, following survey methods developed by Burns et al. [47] for underwater three-dimensional SfM reconstruction surveys [48,53,54,90,122,123]. A Sony α7r IV with a 12–24 mm f/2.8 lens set at 24 mm with Easydive Leo3 Wi underwater housing was used for image acquisition. Owing to logistical constraints, an average of 2,500 images per transect were collected in 2022 while an average of approximately 1,200 images per transect were collected in 2020. Images were processed in Agisoft Metashape v2.0 [126] to generate three-dimensional models and orthomosaics of each transect using the processing parameters indicated in Table A in S1 Text, and further details are described in the supplemental methodology. SfM processing with Agisoft Metashape was performed through Ibex, the HPC (High-Performance Computer) cluster at King Abdullah University of Science and Technology (KAUST, Saudi Arabia). All benthic analyses were conducted using the orthomosaics.

### Sea surface temperature data

Two satellite datasets for heat stress were obtained from the National Oceanic and Atmospheric Administration (NOAA) Coral Reef Watch (CRW) and National Aeronautics and Space Administration (NASA). First, monthly SST averages for October 2020 and 2022 were obtained using the NASA MODIS Giovanni Tool at a resolution of 4 km [127], and the 4 km × 4 km pixel value was extracted for each site each year using the multi-values to points tool in ArcMap v10.8.2. While this is the highest-resolution dataset available for the region to date, all the sites were at least 11 km apart; therefore, a site-specific SST for each transect could be estimated. Second, DHWs were obtained from NOAA Coral Reef Watch [112] to visualize and compare the duration and intensity of heat stress for the two years. To corroborate the satellite data, in October 2020, a WTW Profiline 2EA312 pH/Cond 3320 portable meter was used to obtain the SST at each site. In October 2022, an Ocean Seven 310 multiparameter CTD was deployed at each site, and the single surface value was extracted for consistency with the WTW meter measurements. SST data were averaged across all sites and compared between years using Wilcoxon signed-rank tests for both satellite and *in situ* data [128].

### Coral colony fate tracking

Similar to other SfM studies that select a subset of coral colonies per transect [87–91], *in situ* benthic survey methodology currently used in the Red Sea by Red Sea Global [129] was adapted to randomly tag and fate track a subset of coral colonies per transect within the orthomosaics using ArcGIS Desktop v10.8.2 [130]. For each transect, 100 colonies that appeared in both years were digitally tagged and identified to the lowest possible taxonomic level (typically genus) in collaboration with taxonomic experts for reef-building corals and soft corals, and the growth form and condition at T_0_ (October 2020) were recorded (Table 2). For the most abundant taxa (*Pocillopora, Acropora, Goniastrea*, and *Porites*), 15 colonies per genus for each transect were targeted. In cases where model distortion impaired identification, raw images were consulted. Tagged colonies were then located in the T_1_ (October 2022) orthomosaic, and their condition was again annotated (see ESM_1).

**Table 2:** Possible colony bleaching scenario codes with conditions for 2020 and 2022. The scenarios observed in our study in the central Red Sea are in bold. The first letter of the pair in each code describes the condition of the colony in 2020, and the second letter describes the colony in 2022. For example, a colony with the code ‘BA’ refers to a colony that exhibited partial bleaching in 2020 and normal coloration in 2022. The colors indicate simplified categories that were used in binomial logistic regression analyses and correspond to the colors in Fig 5.

		2020					
		Normal	Partial bleaching	Complete bleaching	Partial mortality	Partial bleaching & partial mortality	Complete bleaching & partial mortality
2022	Normal	**AA**	**BA**	**CA**	DA	EA	FA
	Partial bleaching	AB	**BB**	**CB**	DB	EB	FB
	Complete bleaching	AC	BC	**CC**	DC	EC	FC
	Partial mortality	**AD**	**BD**	**CD**	**DD**	**ED**	**FD**
	Partial bleaching & partial mortality	**AE**	**BE**	CE	DE	**EE**	FE
	Complete bleaching & partial mortality	AF	**BF**	**CF**	DF	EF	FF
	Complete mortality (present)	**AG**	**BG**	**CG**	**DG**	**EG**	**FG**
	Complete mortality (absent)	**AH**	**BH**	**CH**	DH	**EH**	**FH**

**Fig 2:**
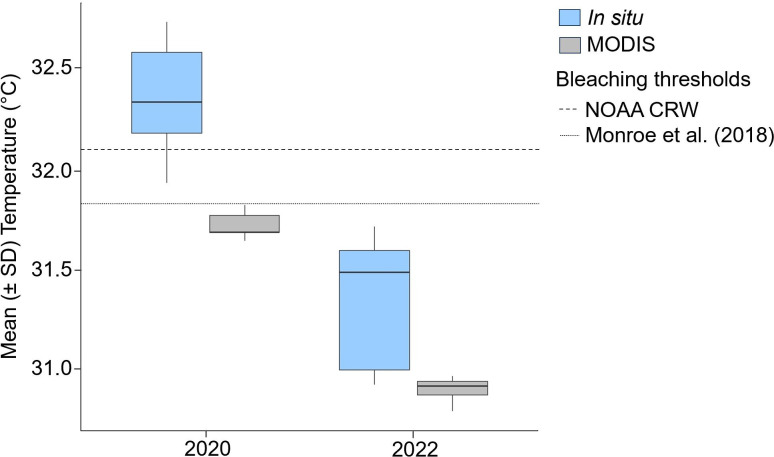
Average (± standard deviation) sea surface temperature (°C) across the seven study sites in the central Saudi Arabian Red Sea for *in situ* measurements (light blue) and MODIS data (gray) [127]. *In situ* data were collected with a WTW meter in 2020 and with CTD OceanSeven in 2022. Bleaching thresholds described for the region by NOAA Coral Reef Watch and Monroe et al. [63] are indicated by dotted lines.

**Fig 3:**
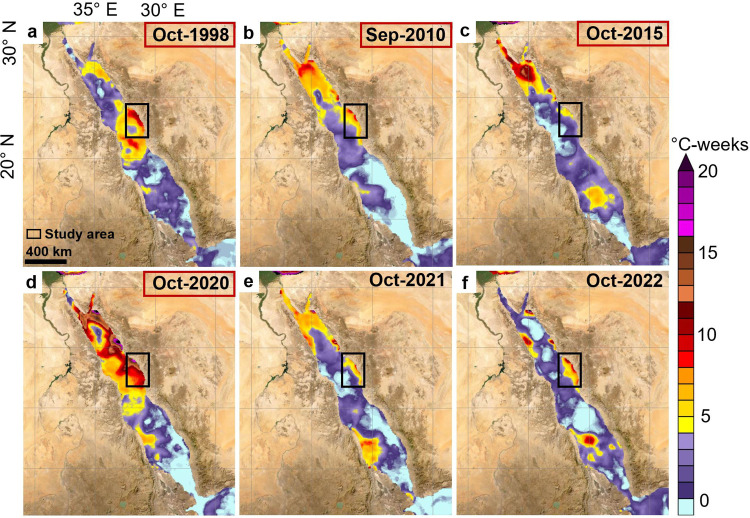
Degree Heating Weeks (DHW) for the Red Sea from October 1998 to October 2022 (a-f). The black polygon includes the study region in the central Red Sea between Rabigh and Yanbu. Red polygons highlight bleaching events in (a) October 1998, (b) September 2010, (c) October 2015, and (d) October 2020 [131]. Basemap satellite images accessed from World Imagery ESRI Tile Layer. Credits: Esri, Maxar, GeoEye, Earthstar Geographics, CNES/Airbus DS, USDA, USGS, AeroGRID, IGN, and the GIS User Community.

**Fig 4:**
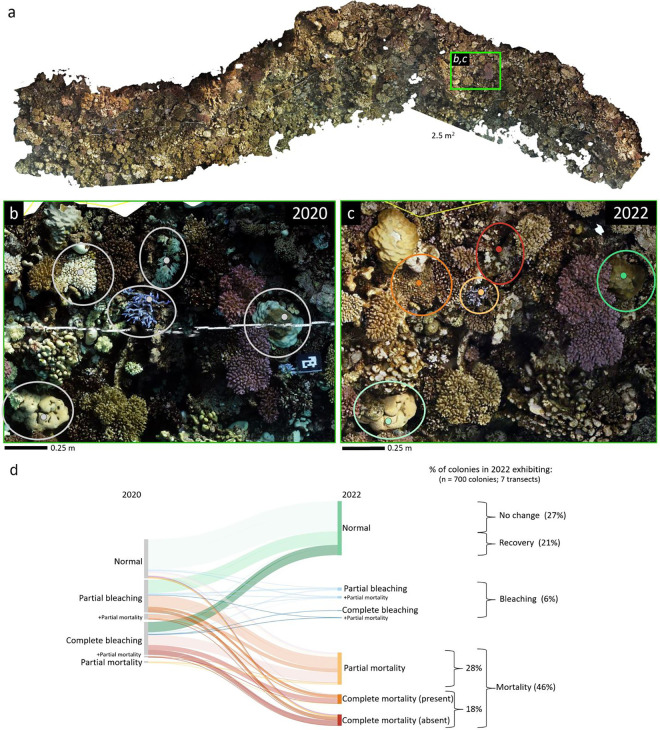
Observed coral bleaching scenarios in the central Saudi Arabian Red Sea from October 2020 to October 2022. (a) Example orthomosaic of site Blank 2 in 2022. The green polygon indicates a 2.5 m^2^ area along the transect, where five colonies illustrate example scenarios in (b) 2020 and (c) 2022. The colors of the circles correspond to different fates in 2022: no change (light green), recovery (dark green), partial mortality (yellow), complete mortality with the skeleton present (orange), and complete mortality with the colony absent (red). (d) Sankey diagram summarizing all the observed coral bleaching scenarios in 2020 (left-hand side) and 2022 (right-hand side) for the 700 hard and soft coral colonies surveyed across the seven sites. Line thickness is proportional to the number of colonies that exhibited a particular scenario.

The condition (state of the colony at a given time point) for 2020 and 2022 was coded by the scenarios described in Table 2. To streamline the analyses, paling and fluorescing colonies were classified as partial bleaching. Partial mortality in 2022 was defined as a qualitative negative change in live coral tissue. The remaining living tissue of the colony otherwise had normal coloration. The scenario refers to the condition of the colony across both years. Scenarios were then grouped according to the changes exhibited over the study period via the adapted categories discussed in Baird and Marshall [11]. The category refers to the change exhibited between the two years and includes (1) no visible change in coloration during the study period, (2) recovery (bleaching in 2020 and normal coloration in 2022), (3) bleaching in 2022, (4) partial mortality of the colony in 2022, (5) complete mortality (with the skeleton present), and (6) complete mortality (colony is absent from the transect).

To understand community composition across transects, the point-intercept methodology was applied to the 2020 orthomosaics. Overall benthic categories were identified every 0.5 m along three replicate 20 m long transects per orthomosaic.

Statistical analyses were run in R v4.3.1 [132] to determine the prevalence of bleaching in 2020 and the fates of colonies in 2022. First, all colonies were pooled across sites, and all scenarios were summarized in a Sankey plot to understand the overall severity of bleaching and mortality of the 2020 bleaching event. To describe overall bleaching and mortality patterns for taxa represented by at least ten [133] colonies across all the sites in total, the scenarios were first simplified to two categories each for 2020 (not bleached and bleached) and 2022 (survival and mortality) to maximize the sample sizes per category and enable binomial logistic regression analyses. The color coding in Table 2 shows how the scenarios were simplified. A binomial logistic regression was conducted via the R package stats v4.3.1 [132] to determine whether bleaching patterns differed across sites and taxa. A second binomial logistic regression was conducted to determine if mortality differed across sites and taxa. For both models, a family quasibinomial was used to account for overdispersion, and residual plots were examined. Tukey’s post hoc test for pairwise comparisons was conducted via the R package multcomp v1.4-25 [134] to determine which sites and taxa were significantly different. Finally, a subset of taxa that met at least one of the following criteria was selected to be categorized into winners and losers (*sensu* [51]): (1) the taxon was represented by more than 40 colonies and was significant in the binomial logistic regression analyses, or (2) the taxon has previously been reported as a winner or loser in the Red Sea and other geographic regions [13,51,52,55,63,93]; these taxa were *Porites, Montipora*, *Pocillopora, Goniastrea, Acropora*, *Stylophora, Sclerophytum,* and Xeniidae. Those colonies were pooled across sites, and scenarios were summarized in Sankey plots for each taxon. Losers were defined as those taxa that (1) exhibited moderate to high complete mortality rates (≥20%) [20] and/or (2) exhibited little recovery (<20%), with recovery defined as colonies that exhibited partial or complete bleaching in 2020 and normal coloration in 2022. All Sankey diagrams were generated using the online tool https://sankeymatic.com/build/. Finally, nonmetric multidimensional scaling (NMDS) analysis was performed via the R package vegan v2.6-4 [135] to ordinate hard coral taxa according to their proportions of different bleaching scenarios outlined in each Sankey plot.

To address the aims of this study, we used a consistent conceptual framework in which we defined four key bleaching terms: resistance, tolerance, susceptibility, and resilience. Resistance refers to colonies that exhibit lower bleaching than adjacent colonies exposed to the same thermal stress [20,93,136] and can be described by the prevalence of bleaching. Tolerance is when bleached colonies recover, and mortality is lower while survival is higher than adjacent colonies exposed to the same degree of thermal stress [20,136]. The susceptibility of coral colonies to bleaching then encompasses both resistance and tolerance collectively or whether the coral bleaches and suffers mortality after being exposed to thermal stress [20,136]. Resilience is at the larger, ecosystem scale and refers to the ecological capacity of a reef ecosystem to recover following a large-scale bleaching event and persist at a steady state in terms of species diversity and relative abundance [20,136,137].

## Results

### Sea surface temperature

SST across sites was significantly greater in 2020 than in 2022, according to both *in situ* measurements and satellite data (p value = 0.007 each; Fig 2). On average, *in situ* values were approximately 1 °C higher in 2020, and satellite SSTs were about 0.8 °C higher in 2020. For reference, a historical annual baseline for the region is provided in Fig A in S1 Text. In 2020, during the bleaching event, *in situ* temperatures ranged from 31.9 °C to 32.7 °C, whereas satellite temperatures ranged from 31.66 °C to 31.79 °C (Table B in S1 Text). Furthermore, DHWs were anomalously high in October 2020, reaching at least 12 °C-weeks in some areas [112], which is 5 °C-weeks higher than the maximum reported in the region in 2010 [107] (Fig 3). Additional maps of the study area showing the DHW for each year are presented in Fig B in S1 Text.

### Orthomosaic properties

A total of 25,865 digital images yielded 14 SfM orthomosaics for the seven transects surveyed in 2020 and 2022. Since transects were established along a depth contour, most orthomosaics exhibited a curved shape, following the shape of the reef. The models were not aligned due to differences in image acquisition between years, which resulted in slightly different angles and greater errors in the 2020 models. Nevertheless, a qualitative visual assessment indicated that at least 70% of each of the seven transect areas sufficiently overlapped between years, allowing for identification and matching of 100 colonies per transect for each time point. The final area of overlap between years was, on average, 64.2 m^2^ across transects. The average root mean square error (RMSE), a measure of reprojection error [138] for the 2020 orthomosaics was 0.032 m ± 0.031 m, whereas the average RMSE for the 2022 orthomosaics was 0.012 m ± 0.013 m. The average resolution for the 2020 orthomosaics was 0.52 mm ± 0.25 mm, and that for the 2022 orthomosaics was 0.61 mm ± 0.09 mm.

### Coral colony fate tracking

Across the seven transects, a total of 700 coral colonies representing 35 taxa were digitally tagged, identified, and annotated for 2020 and 2022 (Fig 4a–c). In total, 65% of the colonies exhibited partial or complete bleaching in 2020, and nearly half of the colonies exhibited partial or complete mortality in 2022, although 18% exhibited complete mortality (Fig 4d). Approximately 6% of the colonies were bleached again in 2022. Finally, about 27% of the total colonies showed no visible change in coloration over the study period, while 21% showed recovery over the two years. Bleaching prevalence across sites ranged from approximately 50% of colonies to nearly 80%; however, there was no clear spatial pattern, and there were no significant differences in bleaching patterns among sites (Fig C in S1 Text; p value = 0.0695). The overall mortality rates ranged from 36% of colonies on Merulina Reef to 61% on Blank 2, and only these two sites were significantly different (p value = 0.0059). Overall benthic community composition varied across sites, and Merulina Reef had the most unique coral community (Fig F in S1 Text).

**Fig 5:**
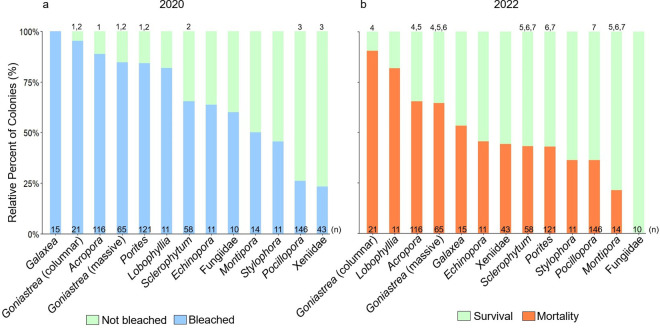
Percent of coral colonies in the central Saudi Arabian Red Sea that were (a) bleached (blue) and not bleached (green) in 2020 and (b) exhibiting survival (green) and mortality (orange) in 2022. Numbers above bars indicate significantly different groups based on binomial logistic regression analyses, and taxa without numbers above were statistically similar to the rest. Groups 1 and 2 had significantly different bleaching rates, and Groups 3, 4, 5, and 6 had significantly different mortality rates.

In total, 13 coral taxa were represented by at least ten colonies. Of these, *Galaxea, Goniastrea, Acropora, Porites, Lobophyllia, Sclerophytum, Echinopora*, and Fungiidae had the highest prevalence of bleaching, whereby more than 50% of colonies across sites were bleached in 2020 (Fig 5a). *Montipora* and *Stylophora* presented a bleaching rate of approximately 50%. Relatively low incidences of bleaching occurred in *Pocillopora* (26%) and Xeniidae (23%). However, all pairwise comparisons of bleaching across taxa revealed that groups that were significantly different were *Acropora, Goniastrea* (massive and columnar), and *Porites* (Group 1); *Goniastrea* (massive and columnar), *Porites*, and *Sclerophytum* (Group 2); and *Pocillopora* and Xeniidae (Group 3) (Table C in S1 Text; p values < 0.05). By 2022, approximately 25% or more of the colonies per taxon exhibited partial or complete mortality, with *Goniastrea* (columnar and massive), *Lobophyllia, Acropora*, and *Galaxea* exhibiting more than 50% mortality (Fig 5b). *Echinopora,* Xeniidae, *Sclerophytum, Porites, Stylophora,* and *Pocillopora* exhibited between 25% and 50% mortality, whereas *Montipora* presented less than 25% mortality, and Fungiidae showed 0% mortality. The groups that were significantly different were *Goniastrea* (columnar and massive) and *Acropora* (Group 4); *Acropora, Goniastrea* (massive), *Sclerophytum,* and *Montipora* (Group 5); *Goniastrea* (massive); *Sclerophytum, Porites*, and *Montipora* (Group 6); and *Sclerophytum, Porites*, *Pocillopora,* and *Montipora* (Group 7) (Fig 5b; Table C in S1 Text). Detailed scenarios of all the taxa observed are provided in Fig D and E in S1 Text.

**Fig 6:**
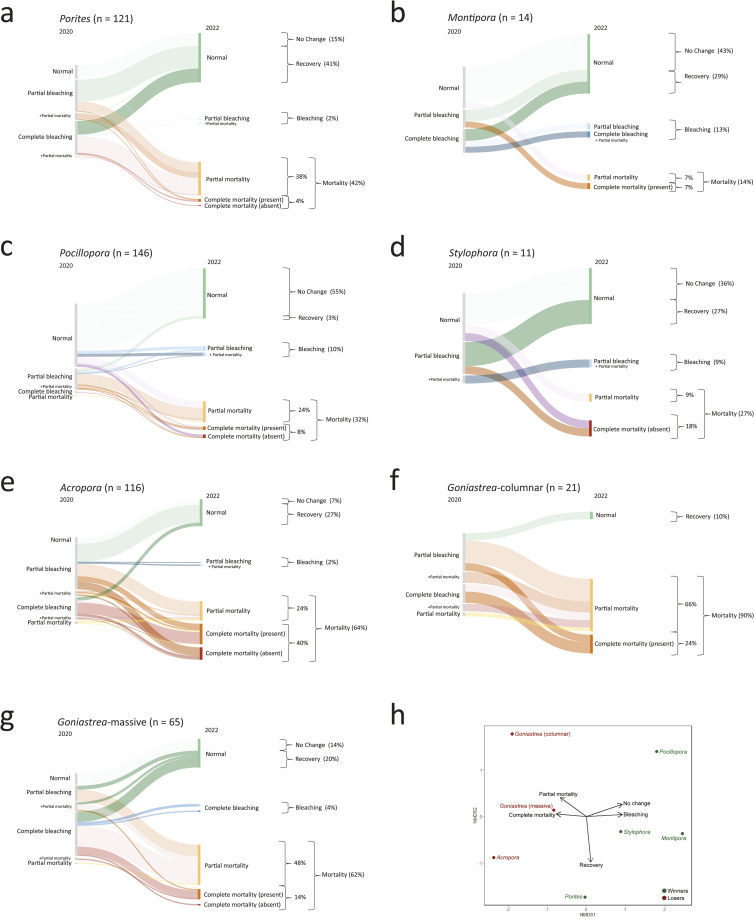
Sankey diagrams summarizing all observed coral bleaching scenarios in the central Saudi Arabian Red Sea in 2020 (left-hand sides) and 2022 (right-hand sides) for (a–d) winner and (e–g) loser hard corals pooled across the seven study sites. Line thickness is proportional to the number of colonies that exhibited a particular scenario. (h) NMDS plot ordinating coral taxa based on their proportion of bleaching scenarios exhibited.

Pooled across the seven transects, 56–72% of the *Porites* (n = 121), *Montipora* (n = 14), *Pocillopora* (n = 146), and *Stylophora* (n = 11) colonies exhibited no visible change in coloration over the study period or recovered over the two years, and less than 20% complete mortality (Fig 6a–d). Each genus also presented unique nuances. For example, *Porites* had a high prevalence of bleaching but also presented a high recovery rate, suggesting high tolerance to bleaching. However, it is also important to note the high level (38%) of partial mortality in *Porites*. Although the sample size was small, the majority of *Montipora* colonies exhibited no visible change in coloration over the study period (43%), and those that did bleach presented a high level of recovery (29%). Furthermore, only 14% of *Montipora* colonies exhibited partial or complete mortality. More than half of the *Pocillopora* colonies exhibited no visible change in coloration over the study period or recovered (58%), and while 24% of the *Pocillopora* colonies exhibited partial mortality, only 8% exhibited complete mortality. Similarly, 63% of *Stylophora* (n = 11) colonies exhibited no visible change in coloration over the study period or recovered, whereas 27% exhibited partial or complete mortality and 18% exhibited complete mortality, although the sample size was relatively small. In contrast, while 34% of *Acropora* colonies presented no visible change in coloration over the study period or recovered, most colonies presented partial or complete mortality (64%), with 40% exhibiting complete mortality (Fig 6e–g). Approximately 90% of columnar *Goniastrea* colonies exhibited partial or complete mortality, with approximately 24% showing complete mortality. Compared with columnar colonies, massive *Goniastrea* colonies had a lower mortality rate (14% complete and 48% partial), but only 20% of the colonies exhibited recovery, while the remaining 18% exhibited no visible change in coloration over the study period or showed bleaching in 2022. The NMDS analysis yielded a stress value of 0.0533 and revealed a separation between winners and losers, as described above (Fig 6h).

**Fig 7:**
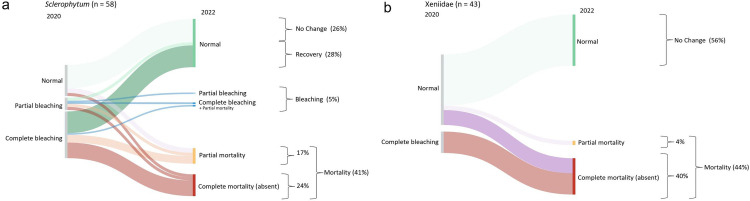
Sankey diagrams summarizing all observed coral bleaching scenarios in the central Saudi Arabian Red Sea in 2020 (left-hand sides) and 2022 (right-hand sides) for loser soft corals (a) *Sclerophytum* and (b) Xeniidae pooled across the seven study sites. Line thickness is proportional to the number of colonies that exhibited a particular scenario.

For soft corals, while 54% of *Sclerophytum* colonies exhibited no visible change in coloration over the study period or recovered, 41% exhibited partial or complete mortality, with 24% exhibiting complete mortality (Fig 7). Similarly, over half (56%) of the Xeniidae colonies exhibited no visible change in coloration over the study period, while the remaining 44% of Xeniidae colonies exhibited partial or complete mortality, with 40% showing complete mortality. Overall, Xeniidae and *Pocillopora* presented the highest rates of resistance (>50%), and *Goniastrea* (columnar) and *Acropora* presented the lowest rates (<10%). *Porites* and *Montipora* presented the highest rates of tolerance (41% and 29%, respectively), whereas Xeniidae and *Pocillopora* presented the lowest rates (<5%). Furthermore, Xeniidae and *Acropora* had the highest rates of complete mortality (40% each).

## Discussion

In this study, the thermal anomaly in the central Saudi Arabian Red Sea during the bleaching event in 2020 was described, and coral colonies were fate tracked to evaluate mortality and survival two years following the bleaching event. This research represents the first record of SfM in the Red Sea and the longest-term change following a bleaching event in some of the most thermally tolerant corals in the world [41,99].

### Sea surface temperature

The concept of thermal thresholds in corals is still a subject of debate, and while intensity, duration, and recurrence of high temperature events are known to influence thermal stress, their respective roles together with irradiance in bleaching are still poorly understood [23,38,39,42]. In this study, although only a 1 °C temperature difference was observed between years, corals presented high mortality rates. This may be in part due to corals living at their thermal maxima in the region [41] but is also likely a function of considerably prolonged thermal stress. Indeed, the estimated 12 °C-weeks reported in the central Red Sea in 2020 was the highest on record in the region at the time of this study to our knowledge. The *in situ* temperature range in 2020 in this study was similar to the temperature range described during the bleaching event in 2015 [63], providing further support for this threshold in the region. However, there is a sparsity of historical *in situ* data, and satellite data are relatively coarse in the central Red Sea; therefore, future studies should deploy a network of data loggers together with monitoring protocols.

### Orthomosaic performance and the relevance of permanent SfM transects for monitoring

SfM methodology enabled a high-resolution, verifiable record of benthic communities, which allowed for the tracking of individual coral colonies across a relatively large spatial scale over two time points. SfM processing using Agisoft Metashape via Ibex resulted in a reconstruction time of 12–16 hours per transect, which was relatively quick compared to an average desktop computer, making this methodological workflow more efficient and scalable. Owing to differences in image acquisition methodology across the two years, our analyses were limited to point-based annotations using orthomosaics and precluded two- and three-dimensional spatial analyses in several ways. First, bleaching and mortality observations were qualitative because changes in surface area could not be accurately quantified at the colony scale. Similarly, structural complexity of each transect could not be accurately quantified and compared across years. Nevertheless, the use of SfM in this study was fundamental to support the first assessment of long-term changes in scleractinian and octocoral colonies following a major reef bleaching event in the central Saudi Arabian Red Sea. Permanent SfM plots surveyed again over time can provide valuable baseline information in the context of increasing bleaching events and other disturbances and enable the ability to investigate additional, unforeseen questions in the future. In fact, two more bleaching events have already occurred in the central Red Sea since 2020 [131,139,140], and a subset of the transects presented in this study has been resurveyed. Furthermore, the methodology outlined here can serve as a reference for further studies. We suggest the implementation of standardized monitoring protocols and prioritizing the establishment of a network of permanent monitoring sites to facilitate the collection of robust, time series data that can enhance our understanding of coral recovery and ecosystem resilience over time.

### Coral bleaching prevalence across sites

The similarity of bleaching patterns across sites could be a result of a number of factors. Although the sites surveyed in this study spanned 165 km of coastline, they were all located within the central Red Sea, which is a region spanning approximately six degrees of latitude [141,142] that is distinct in terms of temperature and other water properties [143], habitat types [94], fish and benthic communities [125,144], and genetic connectivity [145]. In fact, *in situ* measurements and satellite data revealed that temperature anomalies were relatively similar across sites. Furthermore, all the sites were located on offshore reefs, which could have minimized the potential for terrestrial and inshore stressors to influence and compound thermal stress [53,146].

While overall mortality rates were not significantly different across most sites, it is important to note that sites where the *in situ* temperature was more than 1 °C greater in 2020 than in 2022 generally presented higher mortality rates than sites where the temperature anomalies were less than 1 °C. These findings support those of Jokiel and Coles [38], who reported that even 1 °C greater than the summer maxima can lead to mass bleaching events. Interestingly, the only mortality rates that were significantly different were those of Merulina Reef and Blank 2, even though they are adjacent sites (Fig 1). This could have been in part due to differences in benthic community composition (Fig F in S1 Text). The coral community of Merulina Reef was the most distinct across the seven sites and was dominated by *Acropora* thickets. Although *Acropora* is typically classified as a loser, as in this study, the formation of dense thickets may facilitate survivorship through self-shading [147], increased asexual propagation, or minimizing additional pressures, such as predation [148]. Indeed, while the proportion of partial mortality of *Acropora* colonies on Merulina Reef was relatively high, the complete mortality rate was lower than that for *Acropora* colonies at some of the other sites (Fig G in S1 Text).

Although different methodologies were utilized, the range of bleaching prevalence across sites in this study (50–80% of corals) was at the higher end of the range reported during the 1998 (65%) [22], 2010 (13–82%) [93], and 2015 (0.6–67%) [63] bleaching events in the central Red Sea. However, previous studies included more susceptible inshore reefs [63,93], whereas in this study, only offshore reefs were surveyed. Considering the bleaching prevalence for only offshore reefs, bleaching was substantially greater in 2020 (50–80% of corals) than in 2010 (19.6% ± 4.6% of hard corals) [93] and 2015 (2.2% ± 2.7% of hard corals) [63]. Furthermore, the 2015 bleaching event was globally considered to be the longest bleaching event on record [105,106]; however, the incidence of bleaching was actually lower in the Red Sea during this event than in 2020. In addition, the bleaching incidence observed in this study was at the higher end of the range reported from the Great Barrier Reef (20–60% of reef flats exhibited severe bleaching) during the same year [115,116]. The bleaching incidence reported in shallow coastal areas in the South China Sea in 2020 (45–80% of corals) [113] was similar to the incidence reported here; however, local eutrophication in the South China Sea may have contributed to greater thermal sensitivity. However, incidence of bleaching was higher in the South China Sea during the most recent event in 2024 [120].Together with the DHW analysis previously discussed, these findings suggest that the 2020 bleaching event may be the most severe bleaching event on record in the central Saudi Arabian Red Sea at the time of this study, however additional bleaching in the region has been recorded since 2020 [118,119,140]. Importantly, since this event overlapped with the COVID-19 pandemic, few studies have investigated bleaching incidence, and it is possible that impacts in other geographic regions may not have been recorded.

### Bleaching susceptibility across taxa

A total of 700 coral colonies encompassing 35 taxa (32 genera plus two families) were fate tracked, and at least ten colonies per taxon were included in the descriptive logistic regression analyses, whereas at least 40 colonies per taxon with the exception of *Montipora* (n = 14) and *Stylophora* (n = 11) were investigated in the more detailed Sankey plots. With the exception of *Montipora, Porites*, *Lobophyllia,* and Fungiidae, all the taxa represented by at least ten colonies presented complete mortality rates higher than the expected background mortality for the region (7%; Fig 5) [92], suggesting the occurrence of a disturbance beyond inherent dynamics from October 2020 to October 2022. However, it is important to note that this reference background mortality rate was estimated by the percentage of benthic cover represented by dead coral [92], whereas the mortality rate in this study was estimated by the percentage of colonies that exhibited complete mortality over the two years. In this way, mortality estimates in this study are cumulative, including both bleaching-related and background mortality, and mortality rates may have been influenced by additional factors that were not observed, such as disease or crown-of-thorns. Similarly, as an opportunistic snapshot in time, a colony that was observed to exhibit no visible change over the two years could have bleached sometime after the initial survey and recovered prior to the second survey. However, there is an information deficit concerning the seasonal mortality regimes of corals in the central Saudi Arabian Red Sea, which is an important topic for further research.

Across the 13 taxa represented by at least ten colonies, three groups were significantly different and characterized by relatively high and low bleaching; however, in terms of mortality, four overlapping groups were identified. Although *Galaxea, Millepora, Lobophytum*, and Fungiidae presented more extreme values than these groups did for bleaching or mortality, no significant differences were detected, likely as a result of small sample sizes and unbalanced proportions. Furthermore, some taxa presented similar bleaching responses but significant differences in mortality (i.e., *Acropora* and *Porites,* and *Goniastrea* (columnar) and *Porites)*. These findings highlight that patterns of taxa-specific tolerance may be different than those of resistance and underscore the importance of long-term monitoring to investigate both. While overall bleaching rates were lower during the 2010 and 2015 bleaching events in the central Red Sea, *Goniastrea, Porites, Acropora, Montipora*, and *Pocillopora* exhibited similar relative bleaching patterns in 2020 in this study [63,93]. The taxa that were least impacted during the 2020 bleaching event (winners) presented relatively high resistance to bleaching (*Pocillopora* and *Montipora*), relatively high rates of tolerance (*Porites*), or a combination of both (*Stylophora*). The taxa that were most impacted (losers) were those that presented a high incidence of bleaching, high complete mortality rates (≥20%), and/or little recovery (<20%), namely, *Acropora, Goniastrea*, *Sclerophytum,* and Xeniidae.

### Winners and losers: A fluid scenario

*Acropora* (traditionally a loser), *Porites* (winner) and *Montipora* (winner) fell into the expected bleaching categories based on the growth form and results of previous studies, although the sample size was relatively small for *Montipora*. *Porites,* for example, are mainly massive corals and actually exhibited enhanced calcification during and two years following the 2015 bleaching event in the Red Sea, demonstrating the ability to regulate calcifying fluids, which facilitates survival and growth during bleaching [149]. While *Montipora* was previously classified as a loser in the Maldives during the 1998 bleaching event [19,23,100–102], those colonies were generally plating, whereas the colonies in this study were predominantly encrusting, which also supports previous bleaching patterns. Importantly, *Porites* and *Montipora* presented the highest rates of tolerance, which highlights the need not only to quantify the extent of bleaching for these taxa but also to resurvey colonies postbleaching to understand mortality and recovery patterns. In addition, *Acropora* in this study was reported to be a loser, which corresponds to the results of previous studies [13,51,52,55]. Importantly, branching corals such as *Acropora* serve important ecological functions, such as providing food and habitat for other reef organisms, and a shift from branching to massive corals could lead to a loss in habitat type [32,47,63,102].

Other coral taxa, however, did not fall into the expected categories and were not aligned with previous patterns of bleaching. Very little is known about bleaching in soft corals in general, and while some studies have demonstrated that soft corals may monopolize large areas [68,150,151], the consequences of disturbances on communities are unclear. For example, during the 1998 global bleaching event, high rates of bleaching and mortality for *Lobophytum* and *Sclerophytum* were reported in Palau [81] and the Great Barrier Reef [80], whereas *Sclerophytum* was relatively resistant compared with *Lobophytum* in Okinawa [51]. However, during the 2020 bleaching event in the Red Sea, Xeniidae and *Sclerophytum* presented high mortality rates and were classified as losers. Although Xeniidae exhibited one of the highest rates of resistance, these colonies also presented one of the lowest rates of tolerance and highest rate of complete mortality, similar to the 1998 bleaching event in the Great Barrier Reef [68], suggesting that if bleaching of Xeniidae occurs, there is little chance for recovery. In fact, only some octocorals exhibit weedy traits, whereas others have slower growth rates and fewer opportunistic characteristics [68,150]. This suggests that soft corals may not necessarily dominate following a bleaching event. Indeed, a high proportion of Xeniidae exhibited no visible change in coloration over the study period in only one (Merulina Reef) of the seven sites surveyed in this study (Fig G in S1 Text). Similarly, the Rabigh Offshore site was the only site in which *Sclerophytum* did not exhibit mortality.

The relatively high rate of resistance in branching *Pocillopora* was also unexpected considering the tendency of branching corals to be more commonly affected by bleaching and bleaching-related mortality. During the 2010 and 2015 Red Sea bleaching events, inshore *Pocillopora* colonies were severely affected; however, the mid-shelf and offshore colonies were surprisingly resistant. As all the sites in this study were offshore, this does appear to be a pattern in the central Red Sea. Indeed, it has been shown that Red Sea *Pocillopora* actually has a high capacity to adjust photoprotective pathways in response to environmental conditions, which could suggest regional acclimatization [152,153] or support that Red Sea *Pocillopora* is genetically distinct within the Arabian Peninsula [154]. However, there have also been reports of relatively low rates of bleaching in *Pocillopora* colonies in Moorea, French Polynesia [155,156]. Nevertheless, it is important to note that although resistance was high in *Pocillopora*, these colonies also presented one of the lowest rates of tolerance, which suggests that, similar to Xeniidae, once bleached, recovery may be limited. *Stylophora* colonies presented a complete mortality rate of <20%, and the majority of the colonies presented no visible change in coloration over the study period or recovered, although the sample size was relatively small. Importantly, the two most dominant branching corals typically considered susceptible to bleaching (*Acropora* and *Pocillopora*) differed significantly in both the bleaching pattern and mortality rate. These findings are in contrast with previous studies where branching corals were more susceptible to bleaching than massive forms [13,19,51,63,93,152,153], suggesting that this paradigm may not be universally applicable and potential exceptions under specific environmental conditions may warrant further research as previously suggested [157,158].

Both columnar and massive growth forms of *Goniastrea* fell into the loser category, with the massive form only marginally classified as such. Columnar colonies presented a high mortality rate, and while massive colonies presented a rate of complete mortality <20%, the incidence of bleaching was high, and the recovery rate was only 20%. During previous bleaching events in the Red Sea, *Goniastrea* had a relatively high prevalence of bleaching, and high mortality rates in *Goniastrea* were reported in the Maldives following a bleaching event [13,63,93]. The high rates of bleaching prevalence and mortality and low chance of recovery in massive *Goniastrea* both here and in these studies suggest that massive growth forms may not necessarily be less susceptible to bleaching events [13,19,51,63,93,152,153]. Furthermore, even though both massive and columnar *Goniastrea* fell into the loser category, the scenarios were quite different even within this single genus, which highlights the importance of further research at the species level. Overall, the unexpected and contradictory patterns found in octocorals, *Pocillopora,* and *Goniastrea* in this study highlight the need for a more nuanced examination of growth forms and bleaching patterns. Further research is also needed to understand whether these patterns are geographically unique to the central Red Sea.

### Coral reef ecosystem resilience

With bleaching predicted to become more frequent and severe under future climate change scenarios [23,28,32,121], thermally tolerant Red Sea corals may offer unique insights into coral reef ecosystem resilience. The northern Red Sea is currently considered a thermal refugium for corals and is connected with the central region [32,99,159,160]. As a result of the evolutionary history of Red Sea corals, the SST in the northern region is approximately 4 °C below the corals’ thermal maxima, allowing them to withstand more intense albeit less frequent DHWs relative to those in the southern region [31,161]. For example, while mass bleaching has occurred in the southern and central Red Sea with major impacts previously discussed, the northern region has experienced only minor bleaching since 2007 [32,159,161]. In 2015 in particular, corals in the northern Red Sea did not bleach, despite experiencing greater than 8°C-weeks [159]. It has even been suggested that some Red Sea corals may be capable of long-term adaptation [31,63,93,102,152,153]. However, high mortality rates during recent bleaching events, including the 2020 event reported here, highlight that adaptation is limited [102] as previously suggested in the South China Sea as well [120]. Furthermore, the northern Red Sea refugium may eventually disappear because SST in the region has increased by 0.4–0.45 °C each decade over the past 30 years [32,161].

Assessing the overall resilience of central Red Sea reefs poses a challenge, in part owing to a general lack of data for previous bleaching events. First, both the intensity and duration of elevated temperatures and irradiance influence the severity of a bleaching event [38,39], and both *in situ* and remote sensing data in the region are often insufficient for comparisons of thermal stress across years. Indeed, although DHWs appeared higher in 2020 than in previous bleaching years, *in situ* data to ground-truth past events are lacking. Furthermore, as discussed earlier, previous studies focused on the extent of bleaching and short-term changes in benthic communities. The question then remains as to whether corals are adapting to these events. With long-term *in situ* temperature data, future research could model the relationships among thermal stress, bleaching and mortality patterns to understand the effects across multiple taxa and sites. A larger sample size per taxon would also enable future studies to model probabilities of bleaching and mortality in relation to other factors, such as colony size, energy stores, and symbiont communities. This would facilitate investigations of both resistance and tolerance across multiple spatial scales, providing a better understanding of ecosystem resilience in a changing climate.

## Conclusions

Although differential susceptibility of corals to bleaching – and to a lesser extent, associated mortality – has been examined across various temporal and spatial scales, mortality and survival years following a bleaching event in the Saudi Arabian Red Sea remains particularly understudied [92,93]. In this study, SfM methodology enabled high-resolution fate tracking of individual coral colonies across a relatively large spatial scale. Here, we demonstrated that the 2020 bleaching event may have been the most severe on record in the central Red Sea at the time of this study. The taxa-specific responses highlight the important role of identity in bleaching dynamics and illustrates the need to consider both resistance and tolerance in bleaching susceptibility. Furthermore, this study raises the question of whether the general relationship between coral growth forms and bleaching susceptibility adequately captures the complexity observed across different taxa in the central Red Sea. Nevertheless, this phenomenon of winners and losers may lead to community shifts and a loss of structural complexity, which can have cascading impacts on the biodiversity of coral reefs [51,93,123] and is presently an area for further investigation. Overall, this study represents the longest-term change following a bleaching event in a historically data scarce region, however whether the reefs in this region exhibit significant resilience has yet to be determined.

## Supporting information

S1 Text Supplemental methodology and figures. (DOCX)

S2 Dataset Dataset. (XLSX)
