# Supplementary material for: Unprecedented 2020 coral bleaching reveals unexpected taxa-specific responses in the central Red Sea
Source: PLoS One. 2025 Sep 9;20(9):e0331235. doi: 10.1371/journal.pone.0331235 (PMC12419625; doi:10.1371/journal.pone.0331235)
Supplement: S1 Text — (DOCX) [file pone.0331235.s001.docx]

**Supplemental Methodology**

*Orthomosaic creation*

Polyvinyl tiles (15 by 15 cm) with unique patterns were used as ground control points during SfM imagery collection. Five tiles were staggered along each transect, and two corners of each tile were manually input as targets in Agisoft Metashape. Scale bars between each of the two corners were added to scale the dense point cloud.

*Colony tracking*

To randomly tag colonies, along each transect at T_0_ (October 2020), 20 random points were input in 1 m^2^ quadrats every other meter in ArcGIS Desktop v10.8.2. Following similar methodology to Fox et al. (2019), for points that fell on live coral, those colonies were digitally tagged and identified to the lowest possible taxonomic level (typically genus) in collaboration with taxonomic experts. To reach a total of 100 colonies per transect, orthomosaics allowed us to then haphazardly tag and annotate additional colonies with a focus on the most abundant taxa (*Pocillopora, Acropora, Goniastrea*, and *Porites*) in collaboration with a taxonomic expert. Colonies were selected regardless of size. Initially only random colonies were tagged for a total of 346 across the seven transects, and 27% exhibited no change over the two years, 19% exhibited recovery, 6% were bleached again in 2022, 30% exhibited partial mortality, and 18% exhibited complete mortality. After the most abundant taxa were prioritized to reach 100 colonies per transect (700 in total), 27% of colonies exhibited no change, 21% exhibited recovery, 6% were bleached again in 2022, 28% exhibited partial mortality, and 18% exhibited complete mortality. Since the proportions per category for the total 700 colonies were comparable to the initial proportions calculated from the random 346 colonies, we inferred that our haphazard selections did not skew results.

**Supplemental Figures**

**Table A.** **Parameters used in Agisoft Metashape for generating three-dimensional models, orthomosaics, and digital elevation models.**

|  |  |
| --- | --- |
| **1. Align photos** |  |
| Accuracy | High |
| Generic preselection | On |
| Reset current alignment | On (during re-alignment) |
| Key point limit | 40,000 |
| Tie point limit | 4,000 |
| Apply mask to | Key points |
| Exclude stationary tie points | On |
| Guided image matching | Off |
| Adaptive camera model fitting | On |
| **2. Build dense cloud** |  |
| Quality | High |
| Depth filtering | Mild |
| Calculate point colors | On |
| Calculate point confidence | On |
| Reuse depth maps | Uncheck |
| **3. Build mesh** |  |
| Surface type | Arbitrary |
| Source data | Dense cloud |
| Face count | 1,000,000 (custom) |
| Interpolation | Enabled (default) |
| Point Classes | All |
| Calculate vertex colors | Check |
| Check reuse depth maps | Uncheck |
| **4. Build texture** |  |
| Mapping mode | Generic |
| Blending mode | Mosaic |
| Texture size | 4069 |
| Texture count | 1 |
| Hole filling | Yes |
| Enable ghosting filter | No |


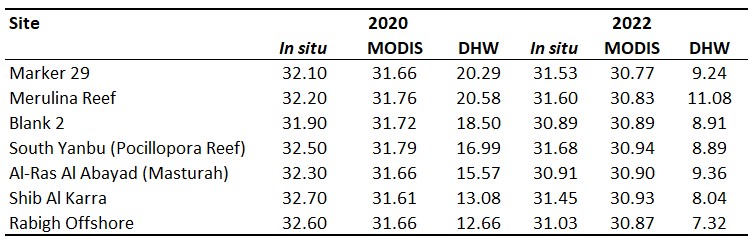
**Table B.** MODIS sea surface temperature data (JPL MUR MEaSUREs Project 2015) and *in situ* temperature (°C) across the seven study sites in the central Saudi Arabian Red Sea in October 2020 and October 2022. *In situ* measurements were collected with the WTW Meter and CTD in 2020 and 2022, respectively.


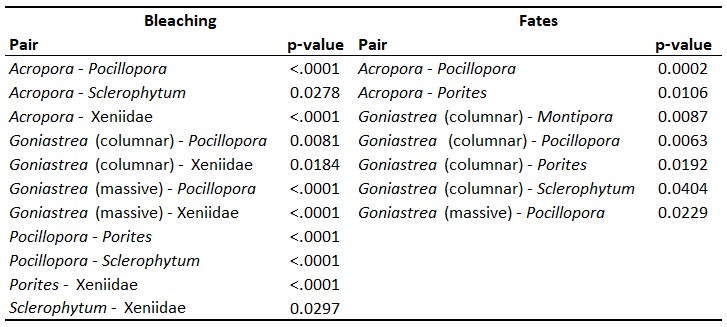
**Table C.** All significantly different pairwise comparisons across coral taxa of colony bleaching rates in 2020 and mortality in 2022 in the central Saudi Arabian Red Sea as determined from post-hoc tests and corresponding p-values.


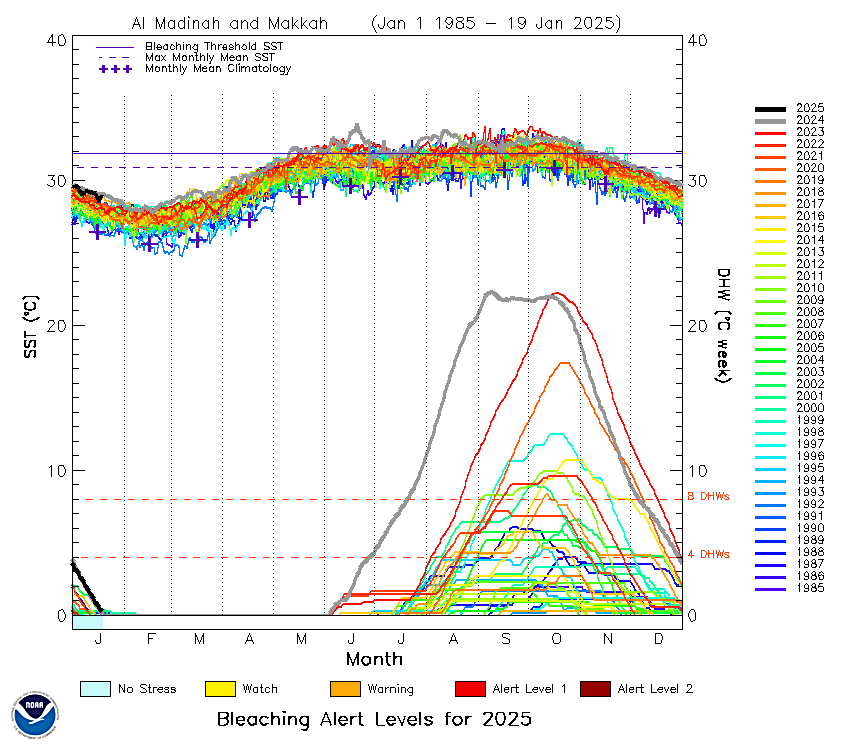


**Fig A.** Historical record of daily global 5km ‘CoralTemp’ sea surface temperature (SST, °C) and degree heating weeks (DHW, °C-weeks) for the Al Madinah and Makkah region that encompasses all seven study sites in the central Saudi Arabian Red Sea from 1985 to 2025. Temperatures were above the bleaching threshold in summer 2020 and DHW exceeded 15°C-weeks. The bleaching threshold (solid blue line) is 1°C greater than the averaged maximum monthly mean. The graph was generated using NOAA CRW Satellite Regional Virtual Station online tool (NOAA CRW)


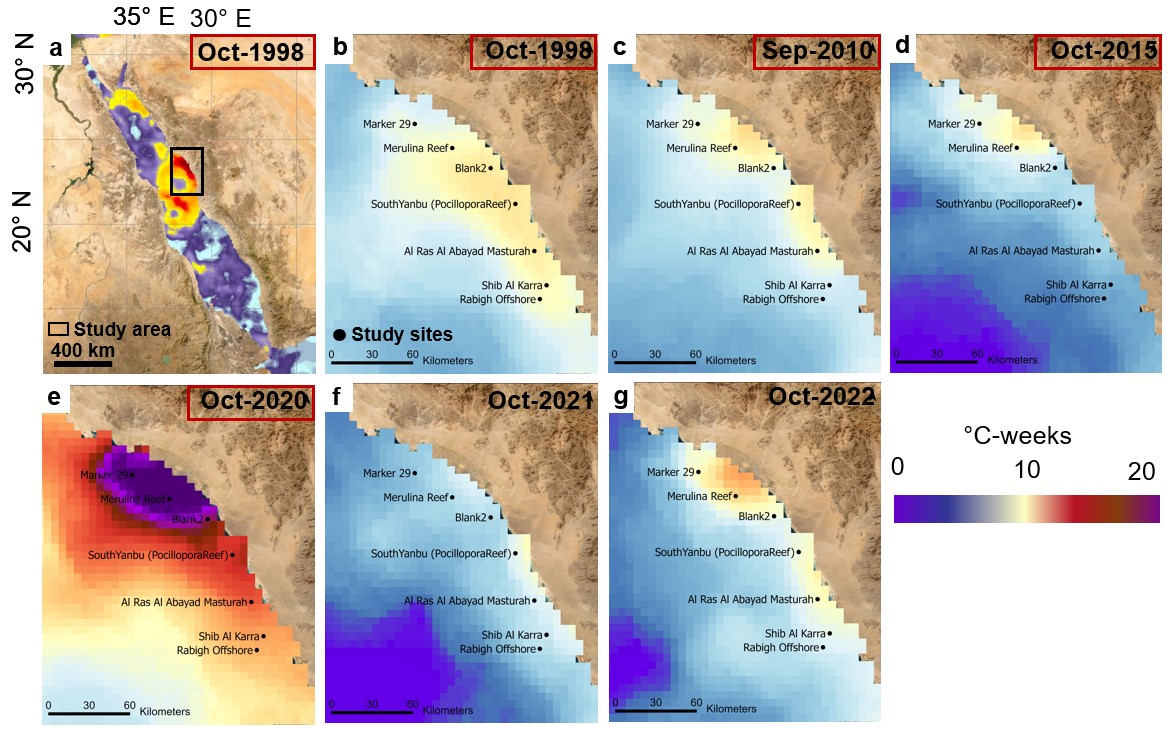
**Fig B.** Degree Heating Weeks (DHW) for the Red Sea in a) October 1998 and b-g) for the study area in central Red Sea from October 1998 to October 2022. The black polygon in a) includes the study region in the central Red Sea between Rabigh and Yanbu. Red polygons highlight bleaching events in a-b) October 1998, c) September 2010, d) October 2015, and e) October 2020 (NOAA Coral Reef Watch). Basemap satellite images accessed from World Imagery ESRI Tile Layer. Credits: Esri, Maxar, GeoEye, Earthstar Geographics, CNES/Airbus DS, USDA, USGS, AeroGRID, IGN, and the GIS User Community


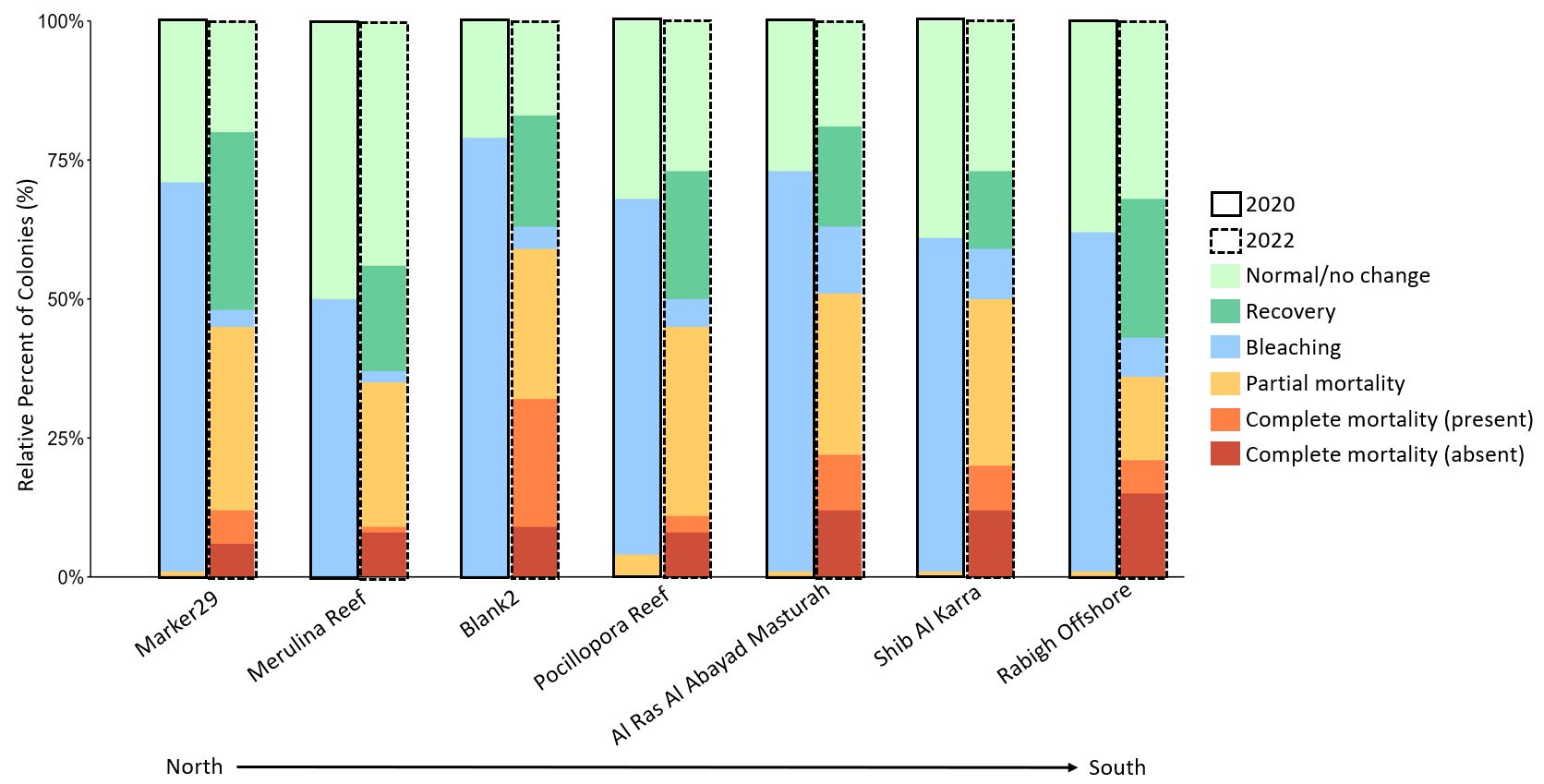
**Fig C.** Percent of colonies that exhibited bleaching in 2020 and each scenario in 2022 by site in the central Saudi Arabian Red Sea


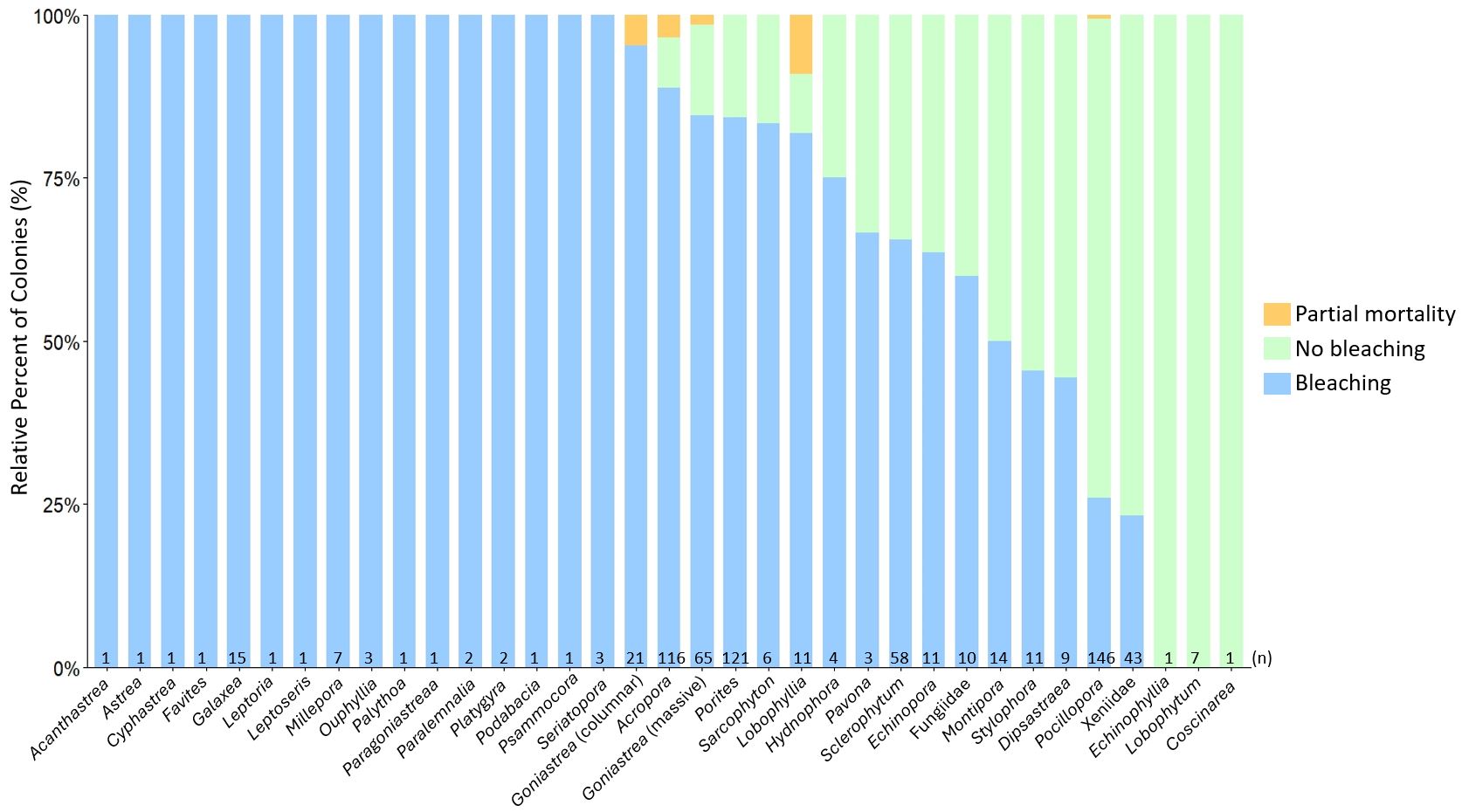


**Fig D.** Percent of colonies that exhibited bleaching in October 2020 for all taxa observed, pooled across all study seven sites in the central Saudi Arabian Red Sea. The total number of colonies tagged was 700, and n shows the total number of colonies for each taxon across study sites

**
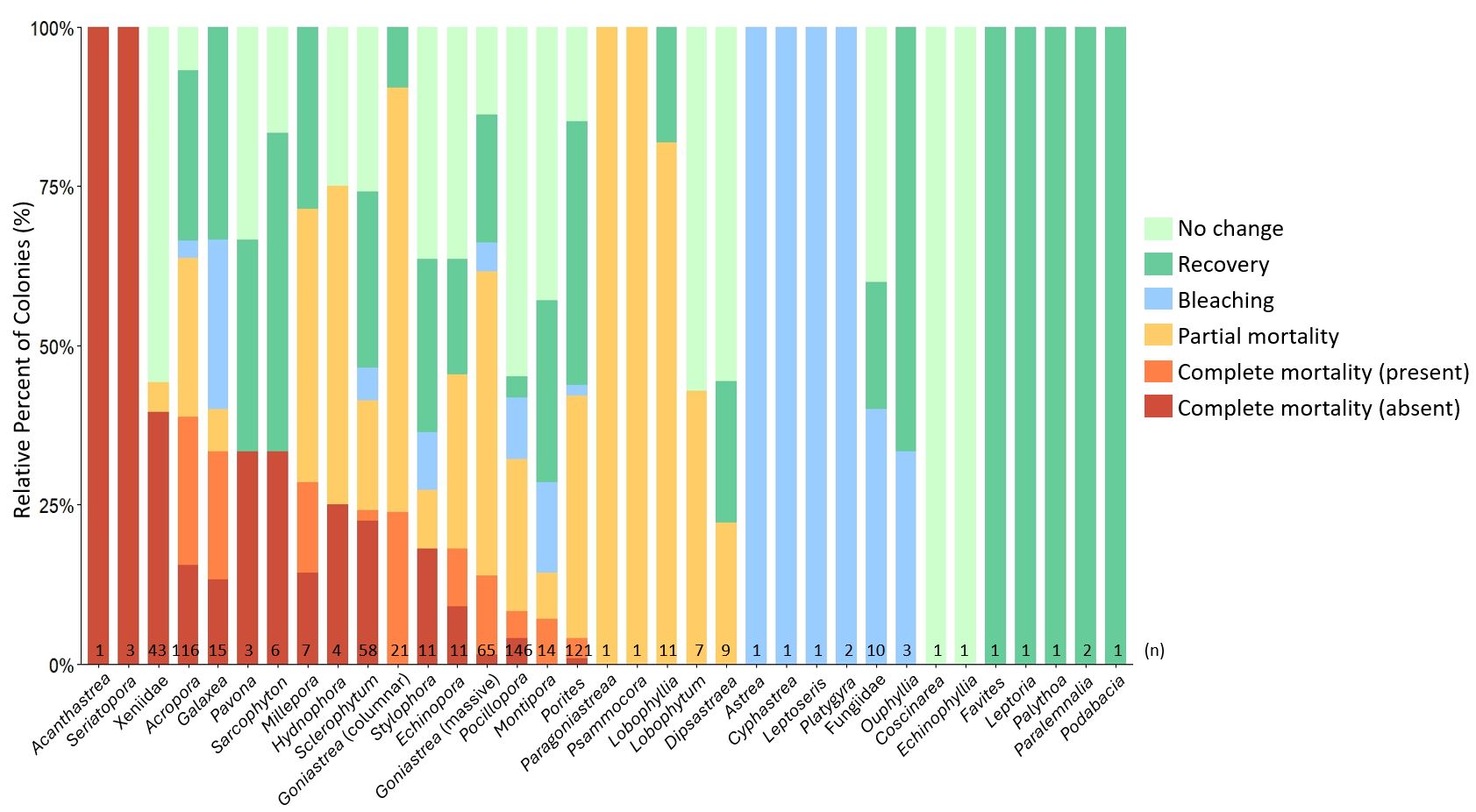
Fig E.** Percent of colonies that exhibited each scenario in October 2022 for all taxa observed, pooled across all seven study sites in the central Saudi Arabian Red Sea. The total number of colonies tagged was 700, and n shows the total number of colonies for each taxon across study sites


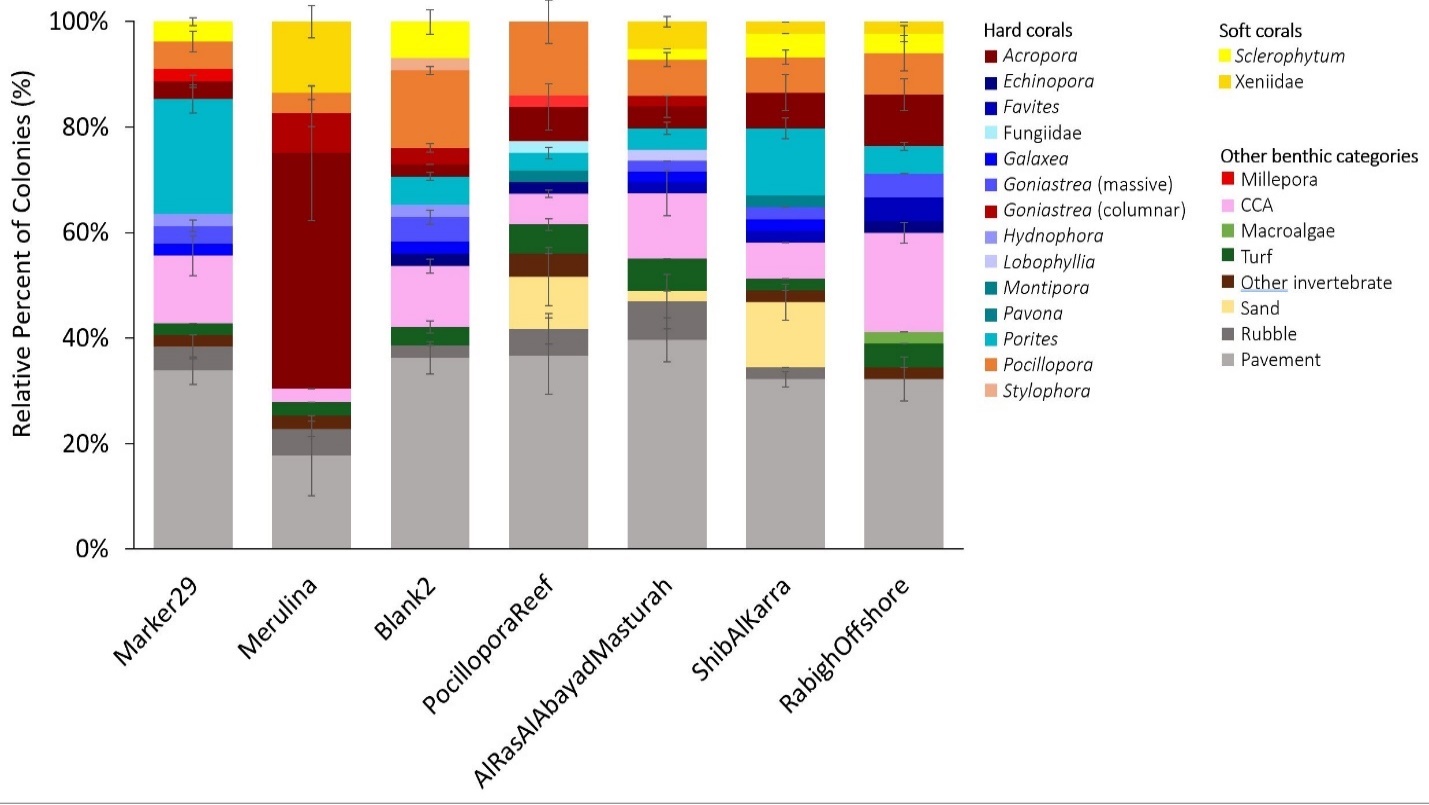


**Fig F.** Benthic community composition by site in the central Saudi Arabian Red Sea in October 2020. Point-intercept transect methodology was used in triplicate to estimate benthic cover for each site using orthomosaics. Error bars indicate the standard error of each category across each site


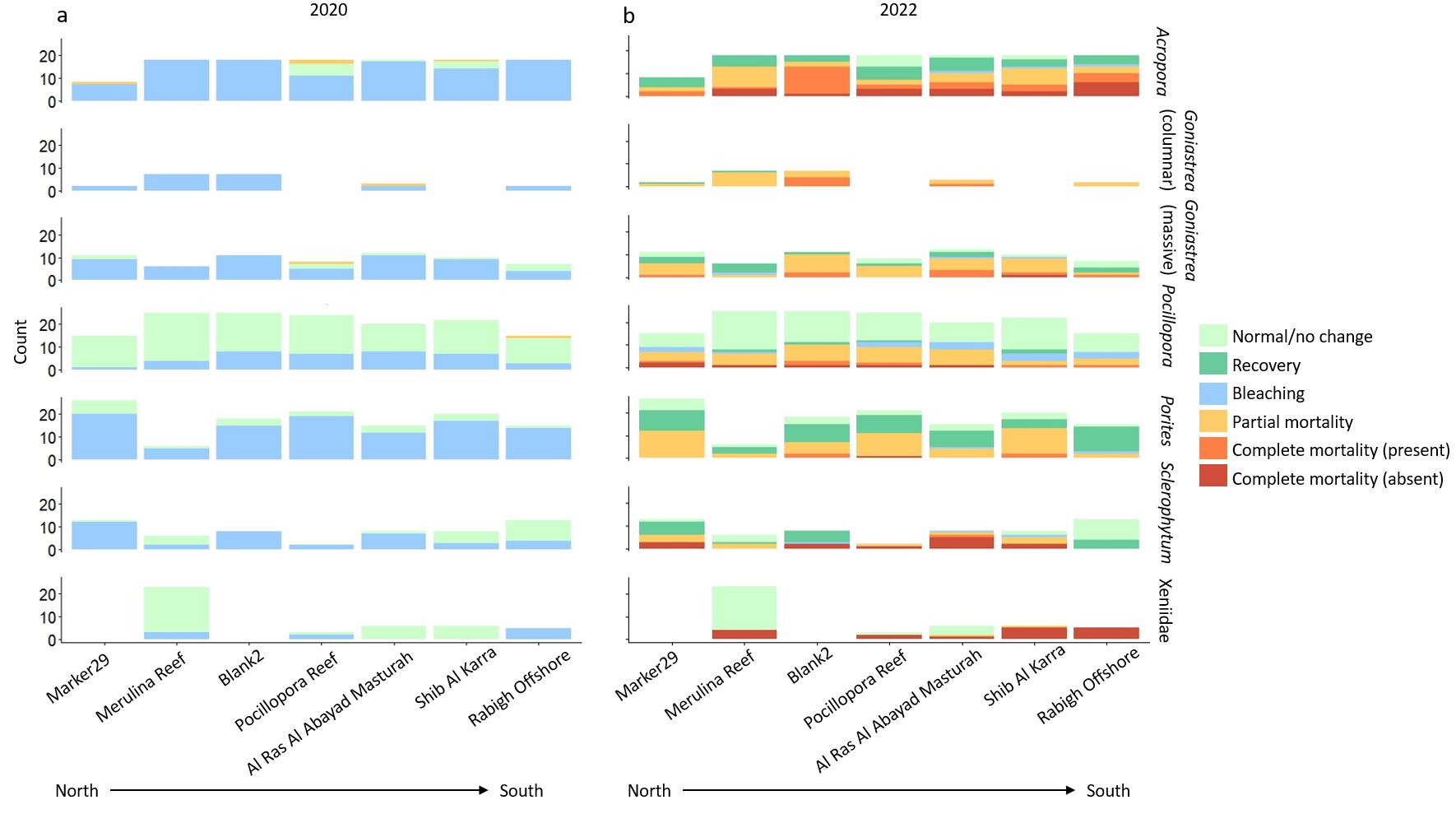
**Fig G.**  Proportion of colonies that exhibited (a) bleaching in October 2020 and (b) each scenario in October 2022 for each abundant taxon per site in the central Saudi Arabian Red Sea
